# Assessment of Infant Exposure to Antidepressants through Breastfeeding: A Literature Review of Currently Available Approaches

**DOI:** 10.3390/pharmaceutics16070847

**Published:** 2024-06-22

**Authors:** Leah Arbitman, Shirley Chen, Brian Kim, Melinda Lee, Peng Zou, Bennett Doughty, Yanyan Li, Tao Zhang

**Affiliations:** 1 School of Pharmacy and Pharmaceutical Sciences, SUNY-Binghamton University, 96 Corliss Ave, Johnson City, NY 13790, USAbkim101@binghamton.edu (B.K.);; 2 Daiichi Sankyo Inc., 211 Mount Airy Road, Basking Ridge, NJ 07920, USA

**Keywords:** antidepressants, breastfeeding, depression, lactation, M/P ratio, nursing infant

## Abstract

Despite the prevalence of depression in lactating mothers, there is a lack of knowledge about the excretion of antidepressants into breast milk and its potential adverse effects on infants. This creates concern, making depressed lactating mothers more likely to avoid pharmacological treatment. Clinical lactation studies are the most accurate and direct method to predict and demonstrate the excretion of antidepressants into human breast milk, and results from clinical studies can be included in drug labels to help physicians and patients make decisions on antidepressant use during lactation. However, there are limited clinical trials and studies on the pharmacokinetics of antidepressants in lactating women because of a lack of enrollment and ethical and confounding factors, creating a lack of knowledge in this area. To bridge this gap in knowledge, alternative methods should be sought to help estimate the antidepressant concentration in breast milk, which is used to assess the safety and transfer of antidepressants into breast milk. We provide a comprehensive review of the usage of these cost-effective, time-efficient, and ethically feasible methods that serve to provide a valuable estimation of the safety and transfer of antidepressants into breast milk before conducting clinical studies.

## 1. Introduction

Women are about two times more likely than men to be diagnosed with depression [1]. Specifically, depression is prevalent in pregnant and postpartum women. It is estimated that 9% to 16% of pregnant women suffer from perinatal depression, and that 14.5% of women suffer from postpartum depression (PPD) [2,3]. 

Depressed mothers are likely to avoid pharmacological treatment due to concerns over adverse effects on infants while nursing. In fact, mothers who take selective serotonin reuptake inhibitors (SSRIs) are 60% more inclined to discontinue breastfeeding compared to mothers who do not take SSRIs. There is a knowledge gap on the effects of antidepressant use on infants during lactation [4]. In a few cases, antidepressant use during lactation was associated with irritability, lack of sleep, and irregular feeding patterns in infants [5,6]. Furthermore, neonatal withdrawal symptoms have also been reported in a few cases involving the use of tricyclic antidepressants [7]. However, leaving PPD untreated can have disastrous effects on mother–infant relationships and family dynamics [8]. PPD strongly correlates with an increased risk of maternal suicide, preeclampsia, eclampsia, and negative parenting behavior [9]. Additionally, untreated PPD is associated with functional impairment in the mother and an elevated risk of psychopathology in the children [10]. Hence, prioritizing the mental health of the mother is crucial for the well-being of the infant, considering that the potential negative effects of untreated depression can outweigh any potential risks associated with using antidepressants during pregnancy and breastfeeding. However, knowledge on antidepressant secretion into breast milk is necessary to assess the potential risk to infants. 

The American Academy of Pediatrics Committee on Drugs considers antidepressants “drugs in which effects are unknown, but may be of concern in breastfeeding” [11]. Antidepressants may enter breast milk and transfer to infants. The FDA has made efforts to increase the availability of high-quality data on drug excretion into human milk for medications women take while they are pregnant or breastfeeding. In 2019, the FDA published the guidance *Clinical Lactation Studies* to encourage sponsors to conduct clinical lactation studies and provide information to facilitate the conduct of such studies. Though these large-scale efforts are underway, there remains a paucity of quality data at this time. There are several challenges in conducting a dedicated mammary pharmacokinetic study in lactating women. It is difficult to enroll lactating women to conduct a dedicated pharmacokinetic study [9]. There is also a limited market value for sponsors [12], and most of the antidepressants that are on the market do not have quality clinical data available. For these reasons, between 2003 and 2012, only 4.7% of drugs approved in the US had human lactating data on the label, making it difficult to evaluate the risks of drug exposure to infants [13]. 

Factors that contribute to drug transfer into breast milk include milk fat composition and milk pH, along with the drug’s molecular size, lipid solubility, protein binding, and transporters [14]. The fat composition in breast milk typically ranges between 35–40 g/L, but varies depending on the phase of milk production, time of day, whether it is foremilk or hindmilk, and even how it is expressed from the breast [14]. Additionally, milk fat can accumulate lipid-soluble drugs which increases the concentration of the drug in the breast milk [14]. The pH of breast milk can range from 6.75–7.42. As mentioned previously, weak-based drugs can concentrate in the milk due to the slightly acidic nature of breast milk [14]. Approximately one week postpartum, drugs with a molecular weight of less than about 200 daltons can pass through pores between mammary epithelial cells into the breast milk while larger nonionized drugs are able to pass through the membrane separating plasma from breast milk by passive diffusion [14]. Maternal inflammatory conditions such as mastitis can disrupt the membrane and allow drugs to pass into the breast milk in greater amounts [14]. Protein binding may also impact drug transfer into breast milk. Breast milk has 8–9 g/L of protein compared to 75 g/L of protein in plasma [14]. In addition to the lower levels of protein, the milk proteins do not bind drugs well; thus, it has been shown that highly protein-bound drugs pass into the breast milk in low concentrations and typically remain in the plasma [14]. Most drugs are passively diffused into the breast milk; however, the breast cancer resistance protein (BCRP) and sodium iodide symporter (NIS) are known to actively transport drugs into breast milk [14]. The amount of breast milk ingested by the infant varies widely. In the first four to five months, milk intake can average approximately 750 to 800 g/day but can range from approximately 450 to 1200 g/day [15]. Past the first four to five months, the amount of breast milk varies even more due to solid foods being introduced into the infant’s diet; thus, it is difficult to standardize the amount of breast milk ingested by a developing infant [15]. Other factors that may affect the infant’s drug absorption through breast milk include the infant’s higher gastric pH, slowed gastric emptying time, reduced bile salts and pancreatic enzymes, underdeveloped drug transporters, developing intestinal microbiome, partial formula feeding, and possibly antibiotic usage [14]. To accurately evaluate the amount of drug transfer and subsequent infant exposure, we must identify and assess each maternal and infant factor.

The lack of knowledge on the extent of the excretion of antidepressants into breast milk and the effects of this on neonatal health is a major issue that dissuades women from breastfeeding despite its many benefits. Breast milk is an exemplary source of ample nutrients and immunological defenses for infants [16]. In fact, infants that are not breastfed have higher mortality and morbidity rates than breastfed infants [17]. Furthermore, breastfed infants have lower risks for diseases such as asthma, diabetes, and hematological cancers [16]. The benefits of breastfeeding are not exclusive to infants. Breastfeeding mothers have more intimate contact and attachment with their offspring and are at a decreased risk for breast and ovarian cancers [18]. Thus, further scientific evidence is needed to ascertain whether it is truly necessary to discontinue breastfeeding when taking certain antidepressants. We provide a comprehensive review of current in vitro and in vivo models for predicting antidepressant excretion into breast milk and discuss pharmacological data on the excretion of antidepressants into breast milk and its effects on breastfeeding infants. 

## 2. In Vitro Models to Predict Drug Excretion into Breast Milk

To determine the safety of breastfeeding while on antidepressants, three factors of drug excretion must be studied: the rate, extent, and distribution of the drug in breast milk. Several in vitro mathematical models have been developed to predict drug transfer into milk, with both strengths and limitations. In vitro models are quite helpful in preliminary studies for determining the extent of antidepressant excretion into breast milk because in vitro studies are less intensive, time-consuming, and costly than in vivo studies and clinical trials. In vitro models must take into account factors such as milk composition, solubility and size of drugs, and milk pH to determine the extent of drug excretion [19]. The milk to plasma concentration ratio (M/P ratio) is often used to estimate an infant’s exposure to medication from breastfeeding. When this ratio surpasses 1, the concentration of medication in breast milk is deemed concerning [20].

The importance of the pH gradient between plasma and milk was first recognized by Rasmussen [21]. There is a pH difference between breast milk and plasma. The pH of breast milk is around 7.1. Plasma is more basic than breast milk, with a pH of 7.4. Weak-based drugs are therefore more likely to diffuse into, and then be trapped in, milk. The unbound distribution model, also named the pH partition model, based on the pH partition theory, uses a rearranged form of the Henderson–Hasselbalch equation to predict the unbound M/P ratio (Mu/Pu) [22]. Atkinson and Begg further improved the pH partition model and developed a phase distribution model with a similar principle to that proposed by Fleishaker et al. [23,24,25]. This model incorporated two additional factors, milk protein binding and milk fat partitioning, to determine drug transfer into milk.

Although these mathematical models and equations are useful for estimating the passage of drugs into breast milk, they are limited because they do not take physiological and some milk composition factors into account. This prompted the development of improved physiological in vitro models that could be used when in vivo studies are not possible. Notarianni et al. developed an in vitro technique to better predict the passage of drugs into breast milk, which can be used as a standard of comparison to in vivo data and determine the effect of milk fat and protein content on the mechanism of drug transfer [26]. This model utilized samples of human breast milk, as well as formula powder and Cow and Gate Plus^®^ baby milk due to their similarities in composition to human breast milk. Plasma was also added and spiked with various concentrations of drugs. There was no difference observed in the rate of drug transfer and M/P ratios among the human breast milk, Cow and Gate milk, and formula milk. This confirmed that the model could work with cow and formula milk and that human breast milk was not needed to determine M/P ratio predictions. Additionally, Notarianni’s model demonstrated the importance of milk composition in determining the rate of drug excretion. As the milk fat and protein content increased, so did the binding and transfer of drugs into breast milk.

Athavale et al., 2013 developed an in vitro cell culture model in order to determine the M/P ratio of therapeutic drugs, including antidepressants [27]. Mouse CIT3 cell lines were used to mimic the behavior and function of mammary alveoli through secretory and tight junctions, respectively. With the exception of salicylic acid, which showed a considerably lower concentration in vitro, the transfer of rifampicin, theophylline, and paracetamol, as reflected by their M/P ratios, showed a similar trend to that seen in vivo.

Zhang et al. recently developed a human mammary epithelium cell (MEC)-based permeability assay to study the excretion of drugs into breast milk and the permeability of the mammary epithelium [28]. This in vitro model used MCF10F cells, which is a human nonmalignant mammary epithelial cell line and formed intact tight junction barriers. A few P-gp transporter drug substrates, including an antidepressant venlafaxine, were utilized as example drugs in this transport assay. This in vitro model estimated that venlafaxine had an M/P ratio of 1.99, which was fairly close to the observed M/P ratio of 2.75.

## 3. Animal Models for Lactation Study

In vivo animal models are also useful to test drug transfer through milk in preclinical studies [29]. Compared with in vitro approaches, in vivo animal lactation models can provide additional insight into the mechanisms of the milk/blood barrier, the effect of parameters such as milk composition and duration of lactation on the rate of medication excretion into milk, and the effects of the excreted drug on offspring health [30].

Multiple factors such as ethics, cost, availability of animals, milk production, milk composition, ease of milk collection, and physiological similarities to humans affect the selection of an animal as an in vivo lactation model [30]. Species used in preclinical studies are non-human primates (NHPs), pigs, dogs, rabbits, and rodents. The hormonal pathways involved in lactation in most of the aforementioned mammals have some similarities to those of humans. Oxytocin stimulates the flow of milk, and prolactin (PRL) plays a major role in lactogenesis [31].

The PRL and oxytocin hormone pathways are similar in humans and animals. In NHPs, oxytocin production peaks during lactation rather than pregnancy, and prolactin (PRL) levels are high during pregnancy to allow mammary tissue to develop for lactation [30]. In minipigs, these hormonal pathways are also similar, since PRL is essential to produce oxytocin for the onset of lactation. However, PRL levels are higher postpartum in minipigs than in humans and NHPs [30]. Dog hormonal pathways are also similar to those of humans, with PRL levels being high during pregnancy and dropping during lactation as oxytocin levels rise [30]. In humans, levels of PRL in the mother greatly rise during pregnancy and contribute to the development of mammary tissue for lactation [32]. After birth, PRL stimulates milk production as levels of progesterone and estrogen decrease [32]. Oxytocin is produced at a faster rate than PRL and stimulates the flow of breast milk [33]. In humans and animals, skin-to-skin contact between mother and baby increases oxytocin levels and therefore milk flow [34]. The similar hormonal pathways make these animal models viable alternatives to clinical studies to predict the transfer of antidepressants into human milk.

## 4. Rodents 

Rodents such as mice and rats are the most realistic option for research laboratory settings due to their ease of handling and housing requirements. However, the use of rodents presents some disadvantages. Rodents produce a very small volume of milk since only one canal per teat is present, making collection very difficult [30]. In terms of milk composition, rodent milk varies greatly from human milk. Rodent milk most notably contains a higher concentration of dry matter, protein, casein, and fat than human milk [35].

Rodent milk collection is time-consuming. Typically, the dose of medication is administered to mothers via oral application. One option is to euthanize the rodent pups and then collect their gastric contents right after milk consumption. Another option is to utilize mini-pumps to collect the milk directly from the mother [29]. The administration of oxytocin to the mother promotes increased milk production [36]. Oxytocin has a very short half-life, so the effects of its administration wear off quickly. Sometimes, even a second injection a few minutes after the first is required to sustain increased milk production [36]. Due to the short half-life of oxytocin, it is not expected that its administration has major effects on the level of drug secretion during lactation. Additionally, while oxytocin can aid in the collection of milk from mice and is necessary for milk ejection during lactation, administering exogenous oxytocin to human mothers has not been shown to increase milk production and therefore drug secretion. One study found that there was no increase in milk production between mothers receiving oxytocin in a nasal spray and the control group [37]. Although more research is needed to confirm this finding, the short half-life of oxytocin and its lack of effect on human milk production demonstrates that the administration of oxytocin is unlikely to greatly affect drug secretion into milk.

## 5. Other Animals

Pigs have genetic and metabolic similarities to humans [30]. There are even pigs produced specifically for biomedical purposes called Göttingen pigs [38]. Compared to other mammals mentioned, pigs produce the most milk due to their large litter sizes of up to 18 offspring [30]. Although pigs represent great candidates for animal lactation models due to their physiological similarities to humans and ease of milk collection, pigs are not used often in a research laboratory setting.

Cow and sheep are other possibilities for animal lactation models. One benefit to using these animals is that they produce large quantities of milk. Cows and sheep produce a higher milk yield than rodents and pigs due to their larger size. Additionally, the composition of cow milk and sheep milk is similar to human milk, although cow milk has a higher content of saturated fatty acids than human milk [35]. Sheep’s milk contains more calcium, sodium, and fat than human milk [35]. A disadvantage to using these animals is that they are large and difficult to deal with in a laboratory environment, and it is also hard to obtain a large sample size of these animals. Very few drug excretion lactation studies have been conducted on cows and sheep. 

In Table 1, the reported animal studies to determine antidepressant drug secretion into breast milk are summarized. Only a few studies measured both milk and plasma concentrations, and thus the M/P ratios were calculated. There was a study which measured drug concentrations in both dam and pup’s serum, and their ratios were calculated and included in the table as well. 

## 6. Human Studies and Reports of Antidepressant Use during Lactation 

After giving birth, mothers are at an increased risk of the onset or worsening of mental illnesses such as anxiety disorders, post-traumatic stress disorder, and PPD [42]. Women who experience mild symptoms are encouraged to seek lower-risk treatments such as psychological interventions; however, pharmacologic treatments are still required for those who show more severe symptoms. Not only so, but the use of antidepressants during pregnancy is closely correlated with adverse events in exposed infants, depending on a variety of factors, including the type of antidepressant taken. In Table 2, the clinical studies/reports with measured antidepressant levels in plasma and milk during lactation are summarized. For some of the antidepressants in Table 2, information on the relative infant dose (RID) is available and therefore included in the table. A RID is the drug dosage that the breastfed infant ingests compared to the maternal drug dosage, written as a percentage [43]. The RID is also known as the weight-adjusted percentage of the maternal dosage, in which the dosage of the drug received by both mother and infant is reported in mg/kg (milligrams of drug per kg of weight). Typically, drugs with a RID of 10% or less are considered to be relatively safe to take when breastfeeding. Caution should be exerted when a drug has a RID between 10% and 25%, and drugs with a RID above 25% are considered unacceptable to take when breastfeeding [43].

Of notable importance is the increase in central nervous system effects such as central nervous excitation and autonomic dysfunction in SSRI-exposed infants. The prevalence of these serotonergic effects is closely linked to maternal SSRI use, and the severity of these adverse effects is said to be congruent with cord blood 5-HIAA levels [44]. In one prospective follow-up study, a statistically significant difference in the serotonergic score was found between SSRI-exposed infants and the control group. Chiefly, those exposed to maternal SSRI use had significantly lower cord-blood 5-HIAA levels, inversely linked to higher serotonergic scores, indicating more severe serotonergic effects.

Another similar study by P Bot et al. describes six infants with neonatal serotonergic symptoms after maternal SSRI use [45]. Namely, the use of sertraline, fluoxetine, and paroxetine, in varying doses, were linked to comorbidities associated with serotonin toxicity or withdrawal, including jitteriness, irritability, hypotonia, and EEG abnormalities. In one such case, the use of paroxetine 20 mg daily at 35 weeks gestation resulted in neonatal jitteriness, myoclonic activity, and feeding problems for 22 days [45]. Likewise, the use of sertraline at 50 mg daily at 32 weeks gestation resulted in smacking of the lips, EEG abnormalities, opisthotonos propensity, and myoclonic activity for 14 days [45].

**Table 2:** Clinical studies/reports with measured antidepressant level during lactation.

Drug	Drug Class ^#^	First-Line/Second-Line Treatment	Study Type	Study Size	Obs. M/P Ratio	RID	Observed Adverse Effects in Infants	Reference
Amitriptyline	Tricyclic	Second line	Cohort study	2 mother–infant pairs	Mean: 0.9	1%	One infant tested in the low normal range for development and was slightly hypotonic	Yoshida et al., 1997 [46]
Bupropion	NDRI	First line	Cohort study	10 mother–infant pairs	Mean: 2.8	0.2%	No adverse effects reported	Kaplan et al., 2004 [47]
Citalopram	SSRI	First line	Cohort study	7 mother–infant pairs	Mean: 1.8	3.2–3.7%	No adverse effects reported	Kristensen et al., 2000 [48]
Clomipramine	Tricyclic	Second line	Cohort study	2 mother–infant pairs	0.4–3.0	1.3%	No adverse effects reported	Smith et al., 1997 [46]
Desipramine	Tricyclic	Second line	Case report	1 mother and her infant	Mean: 1.45	N/A	No adverse effects reported	Reed et al., 1986 [49]
Desvenlafaxine	SNRI	First line	Cohort Study	10 mother–infant pairs	Mean: 2.24	6.8%	7 infants were at a lower growth percentile but all 10 infants had normal development	Teoh et al., 2010 [50]
Dothiepin	Tricyclic	Not available in the U.S.	Cohort study	8 mothers with plasma samples taken from 5 infants	Mean range: 0.78–1.59	0.58%	No adverse effects reported	Lebedevs et al., 1992 [51]
Doxepin	Tricyclic	Second line	Case report	1 mother-infant pair	Mean: 1.66	2.2%	No adverse effects reported	Ilett et al., 1985 [52]
Duloxetine	SNRI	First line	Open label study	6 mother–infant pairs	Mean: 0.25	0.14%	No adverse effects reported	Loghin et al., 2008 [53]
Escitalopram	SSRI	First line	Cohort study	8 mother–infant pairs	Mean: 2.2	3.9%	No adverse effects reported	Hackett et al., 2006 [54]
Fluoxetine	SSRI	First line	Cohort study	23 mother–infant pairs	Mean: 0.62	0.54%	No adverse effects reported	Riggs et al., 2006 [55]
Fluvoxamine	SSRI	First line	Meta-analysis	6 mother–infant pairs	Mean: 0.9	0.98%	No adverse effects reported	Levy et al., 2004 [4]
Imipramine	Tricyclic	Second line	Cohort study	4 mother–infant pairs	Range: 0.9–1.5	2.9%	No adverse effects reported	Smith et al., 1997 [46]
Mianserin	Tetracyclic	Not available in the U.S.	Case reports	2 mother–infant pairs	3.6, 0.8	1.4%, 0.5%	No adverse effects reported	Norman et al., 1993 [56]
Mirtazapine	Tetracyclic	First line	Cohort study	8 mother–infant pairs	Mean: 1.1	1.5%	No adverse effects reported	Ilett et al. 2007 [57]
Moclobemide	MAOI	Not available in the U.S.	Cohort study	6 mother–infant pairs	0.7	1%	No adverse effects reported	Schoerlin et al. 1990 [58]
Nortriptyline	Tricyclic	Second line	Case reports	1 mother–infant pair	Mean: 1.6	1.3%	No adverse effects reported	Skjaeraasen et al., 1988 [59]
Paroxetine	SSRI	First line	Cohort study	9 mother–infant pairs	Mean: 0.6	2%	No adverse effects reported	Touw et al., 2024 [60]
Reboxetine	NRI	Not available in the U.S.	Open-label study	4 mother–infant pairs	Mean: 0.06	2%	No adverse effects reported	Ilett et al. 2006 [61]
Sertraline	SSRI	First line	Cohort study	15 mother–infant pairs	Mean: 2.3	1%	No adverse effects reported	Touw et al., 2024 [60]
Trazodone	SARI	Second line	Clinical trial	6 mother–infant pairs	Mean: 0.14	0.65%	No adverse effects reported	Ross et al., 1986 [62]
Venlafaxine	SNRI	First line	Clinical trial	6 mothers and 7 infants	Mean: 2.5	6.4%	All infants had normal development but 2 had decreased weight gain	Kristensen et al., 2002 [63]

^#^ NDRI: Norepinephrine–dopamine reuptake inhibitor; SSRI: Selective serotonin reuptake inhibitor; SNRI: Serotonin–norepinephrine reuptake inhibitor; MAOI: Monoamine oxidase inhibitor; NRI: Norepinephrine reuptake inhibitor; SARI: Serotonin agonist and reuptake inhibitor.

## 7. Selective Serotonin Reuptake Inhibitors (SSRIs)

SSRIs are the safest antidepressant drug class and are typically the first choice of antidepressant [16]. SSRIs are often prescribed during pregnancy because they are typically better well-tolerated than other antidepressant drug classes [9]. The mechanism of action of SSRIs involves inhibiting the reuptake of serotonin to increase the serotonin concentration in the brain. Serotonin is pivotal for processes such as learning, memory, mood, fear, and social functions [64]. Studies have shown that SSRIs do not easily cross into the breast milk and lead to no increase in malformations during pregnancy [65]. As shown in Table 2, some SSRIs have human M/P ratios less than 1 [4,55,60], while others like citalopram, fluoxetine, and sertraline may go up to more than 1 [48,54,60]. Most drugs penetrate breast milk through passive diffusion. Plasma protein binding can prevent the passage of molecules into breast milk, and since SSRIs are known to have high plasma protein binding values, they are considered the safest option [65].

## 8. Citalopram

In one study on the effects of citalopram on neonatal health, 11 mothers on citalopram during pregnancy and lactation were enrolled, and there was a control group of 10 mothers that were not on drugs [66]. There was no difference in the weights of infants from either cohort. Neurological development of infants from either cohort was also assessed to be identical. The levels of citalopram and desmethylcitalopram in breast milk and serum decreased over time, as well as the levels in infant serum. The individual M/P ratios ranged from 1.2 to 3.3. Depending on the intake volume of milk and absorption of citalopram, infants can have some exposure to citalopram. There was a case report detailing a mother taking 40 mg of citalopram daily during pregnancy and while breastfeeding [67]. The infant had irregular breathing, hypotonia, and irregular sleeping patterns. Twenty-five days after birth, the M/P ratio was 2.0, and fifty-three days after birth, the M/P ratio was 2.8. In another study, seven women taking citalopram and their seven infants were studied [48]. The median dosage of citalopram amongst the seven mothers was 0.36 mg/kg/day. The mean M/P ratio of citalopram was 1.8, with ratios ranging from 1.2 to 3. The mean M/P ratio of demethylcitalopram was also 1.8, with a range of 1.0 to 2.5. The mean RID for citalopram ranged from 3.2 to 3.7%. Citalopram was detected in the plasma of all seven infants, but demethylcitalopram was only detected in the plasma of two infants. Nevertheless, concentrations of citalopram in the infants were low, and none of the infants exhibited adverse reactions. All infants had body weights within the appropriate percentile for their age and tested normally on the Denver developmental screening test.

## 9. Fluoxetine

Fluoxetine has a relatively long half-life, with a single dose ranging between 1 to 3 days. This is an explanation as to why fluoxetine has higher M/P ratios and infant serum concentrations compared to other SSRIs. One study involved 11 mothers taking fluoxetine during pregnancy and breastfeeding and 10 drug-free mothers [55]. In both cohorts, there were no major differences in the weights and neurological development of the infants. Drug levels in maternal serum and milk decreased over time, and the M/P ratios ranged from 0.3 to 2.246. Fluoxetine infant serum levels were highest when the infant was also exposed to fluoxetine in the womb, a phenomenon called prenatal loading [16]. Another study monitored 23 women on a dose of 18.0 to 24.0 mg of fluoxetine daily, 3.7 months postpartum [55]. The M/P ratio of fluoxetine ranged from 0.50 to 0.73, and fluoxetine was detectable in the serum of only two infants after 2 months.

## 10. Paroxetine 

It should be noted that when taken during pregnancy, paroxetine is shown to correlate with an increased risk of heart dysfunction, but since it passes into the breast milk in low concentrations, it still may be safe for lactating mothers to use [3]. In one study, the exposure of six infants to paroxetine at 16 weeks old was determined, and paroxetine was not detectable in any infant serum [68]. The infants had no adverse effects from paroxetine. In another study, the weight and neurological development of 27 infants exposed to paroxetine were compared at 3, 6, and 12 months of age. There was a control group of 19 infants who were not breastfed. The weight of all infants was normal, and the neurological development of all infants except for one was normal. Paroxetine was only detectable in one infant. In a cohort study of nine mothers on paroxetine and their nine infants, paroxetine was undetectable in the plasma of eight infants. The average M/P ratio of paroxetine was 0.6, and the average RID was 2%. These values indicate that there is a lower concentration of paroxetine in milk than in plasma, and that the infants received little exposure to paroxetine through milk [60].

## 11. Sertraline

A pooled analysis of data from 53 mothers was conducted to determine the amount of sertraline that transfers from breast milk to the infant. Researchers found that infants had an average of only 2% of the sertraline plasma levels of the mothers [4]. Another study reported detectable but low serum levels of sertraline in 4 out of 22 infants, with M/P ratios ranging from 0.42 to 4.81 [69]. An additional study investigated 25 lactating mothers taking an average of 82.4 mg of sertraline daily for four months. No adverse effects were reported, and all infants had normal 6-month weight gains [70]. In a cohort study of 15 mothers on sertraline and their 15 infants, sertraline was detectable in all milk samples but was undetectable in the plasma of each infant. The average M/P ratio of sertraline was 2.3 and the average RID was 1%. The concentration of sertraline declined over time in the milk [60].

## 12. PBPK Models to Facilitate the Estimation of Drug Secretion into Breast Milk 

Physiologically based pharmacokinetic (PBPK) models can also be used for estimating the concentration of medication in milk. These models can predict the entire drug concentration–time profile and may be constructed to make quantitative predictions of in vivo drug response as well [28]. Developing PBPK models requires knowledge of the anatomy and physiology of the organism of interest, as well as the physicochemical properties of the drugs to be tested. These models are useful in populations with little clinical study data, such as infant populations. There is variation in the amount of drug excretion into breast milk among drugs. This is because different drugs have different physicochemical properties and transport mechanisms [19]. The composition of milk also affects the amount of drug excretion. PBPK models can be used in tandem with M/P ratios to determine the concentration profiles of drugs in the milk based on the plasma concentrations [19].

The building of a PBPK model to predict drug secretion into breast milk generally follows the following steps. It begins with constructing an adult PBPK model, which is validated with available clinical data. A compartment representing breast milk is then added, and in some cases, an infant PBPK model is further connected to the milk compartment. The physiology, anatomy, and other parameters such as age-dependent enzyme activity are scaled from adult models using established age-dependent algorithms. The ontogeny functions help guide scaling between developmental stages as well. 

Pansari et al. integrated drug and physiological milk properties into the PBPK framework and developed a lactation PBPK model that determines infant daily dose indices [71]. This model can assist with neonatal risk assessment after exposure to drugs that diffuse into the breast milk. The results show that the PBPK model used in this study was effective in predicting maternal plasma and milk concentrations of four basic drugs including the antidepressants of venlafaxine, fluoxetine, and paroxetine.

Maharaj et al. developed a PBPK model and stimulated infant plasma pharmacokinetic profiles after daily exposure to medication via breast milk [72]. The first step was to develop an adult PBPK model utilizing physiological data, physicochemical properties of the drugs, and adult pharmacokinetic data. Examples of physicochemical properties are lipophilicity, molecular weight, pKa, solubility, and oral bioavailability. In the second step, the infant model was developed. While the infant PBPK model has many benefits in determining M/P ratios and area under the curve (AUC) values for drugs, these models are limited by the limited infant plasma data available to verify these models.

Delaney et al. created a PBPK model to simulate the transfer of escitalopram into breast milk and to determine the level of infant drug exposure [73]. The PBPK model was developed by first creating an adult model from available clinical data. If this model did not accurately reflect observed data, parameters were re-evaluated for optimization. The optimized adult model would then be scaled to the pediatric population model. Scaling involved parameters including clearance, protein binding, and overall anatomy and physiology. The PBPK model allowed for successful prediction of plasma AUC levels and showed that infant exposure to escitalopram is generally low. 

Zhang et al. developed a mechanistic lactation PBPK model to predict the human milk concentration–time profiles of transporter-mediated drugs, including the antidepressant bupropion [74]. Predicted pharmacokinetic profiles for the drugs were compared to clinical data of the drugs. The clinical data were derived from a case report of a 37-year-old lactating woman on a 100 mg oral dosage of bupropion for 14 days [75]. The predicted M/P ratio of bupropion was 3.70 with an observed value of 4.44 [74,75]. The purpose of this model is to predict the concentration of these drugs that may diffuse into breast milk with the contribution of drug transporters at different dosages. This, in turn, would allow for prediction of different pharmacokinetics as they exist in breast milk.

Nauwelaerts et al. utilized a generic PBPK model to estimate the concentration of ten different medicines in human milk [76]. PBPK models for “non-lactating” adult individuals were constructed first, and then lactation physiology was incorporated into the models. The models simulated a 3-month postpartum population, and AUC-based M/P ratios and RIDs were calculated from the predicted milk and plasma concentrations. This study selected amoxicillin, caffeine, cetirizine, levetiracetam, metformin, nevirapine, sertraline, valproic acid, tenofovir, and zidovudine as the ten medicines. The lactation PBPK models overpredicted the concentrations of nevirapine and tenofovir in human milk, but reasonably predicted the pharmacokinetic profiles of the other eight drugs. This is a promising model from a safety perspective since there were no concentration underpredictions for any of the drugs in human milk. The models were limited by the lack of clinical data on these medications in lactating individuals. These data would have helped to better assess the predictive performance of these PBPK models.

## 13. Other Approaches

Another in silico technique that works complementary to PBPK modeling is Quantitative Structure-Activity Relationship (QSAR) modeling. QSAR modeling predicts biological activity based on chemical structure and can be used to identify structural features of antidepressants that influence their M/P ratios and enzymatic breakdown. Katritzky et al. developed a QSAR model to predict M/P ratios for a wide array of pharmaceuticals [77]. The model was built by testing the experimental log M/P ratios of the most widely used drugs against theoretical molecular descriptors including topological, constitutional, and thermodynamic. The resulting model showed satisfactory correlation (R^2^ = 0.791) between predicted and observed logarithmic M/P ratios and could, in theory, be used to predict the distribution coefficient between human breast milk and plasma of many different compounds [77]. Maeshima et al. developed a similar QSAR/QSPR model by using a binary classification model to predict M/P_AUC_ ratios [78]. These models incorporated descriptors calculated specifically from the molecular structure of compounds and were found to perform at an acceptable range. A notable disadvantage with QSAR models, however, is that prediction accuracy can be a significant problem due to issues related to data quality, the complexity of the model, and the need for proper validation and testing.

Retrospective analysis of large datasets describing antidepressant exposure in infants and associated outcomes can provide further insight into the risks and benefits of perinatal antidepressant use. Christensen et al. provides a retrospective cohort study to examine the possibility of long-term neurologic deficits in infants exposed to antidepressants [79]. Danish schoolchildren, whose mothers had filled prescriptions for antidepressants during pregnancy, ended up testing 2 points lower on standardized mathematics examinations— a difference found to be statistically significant, but clinically ambiguous. According to a similar retrospective study by Singal et al., in utero exposure to SSRIs or SNRIs was associated with an increase in neurodevelopmental deficits in a cohort of kindergarten children in Canada [80]. These children, whose mothers had filled ≥ 2 antidepressant prescriptions during pregnancy, were found to have increased odds of being developmentally vulnerable, defined as scoring in the bottom 10th percentile of the Educational Developmental Instrument, or EDI [78]. Additionally, children in the exposed group had a significantly higher risk of language and/or cognitive vulnerability [80]. While retrospective approaches are time- and cost-efficient, they carry the risk of biases and cannot be used a priori to run predictions. 

## 14. Challenges

**Figure 1:**
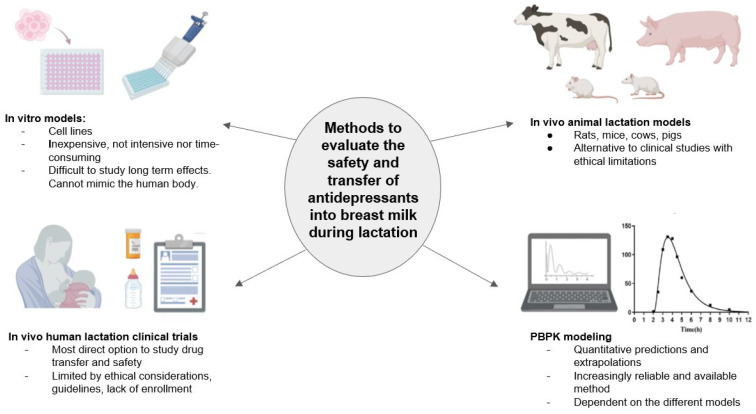
Methods for evaluating the safety of antidepressant transfer into breast milk.

Currently, there are several methods, both in vivo and in vitro, available for evaluating the secretion of antidepressants into breast milk, as depicted in Figure 1. Although many resources are available, there are still gaps in knowledge regarding drug passage into breast milk. Some innate challenges are present. Clinical data showing the effects of antidepressants during lactation largely consist of case reports and studies that have a small sample size, which may not be reliable [16]. If there are detectable levels of a drug in breast milk, researchers must weigh whether it is safe for an infant to consume. 

Human in vivo clinical lactation studies represent the most direct and accurate option to determine the extent of antidepressant secretion into breast milk, as well as any safety risks that these antidepressants pose to infants. However, in vivo clinical lactation studies are difficult because there are numerous ethical considerations and guidelines that must be adhered to. It is also difficult to enroll a significant number of lactating women in clinical studies since there are not always benefits to their participation in said study [12]. Furthermore, with clinical lactation studies, there is the wide amount of variation across different studies in terms of the methodology for collecting and analyzing milk drug concentration data and the timelines of sampling [12]. This creates difficulties in accurately comparing and interpreting data across different studies to make conclusions. The obstacles mentioned above make an antidepressant prescription for lactating women a continuing conundrum.

The recent FDA guidance may encourage pharmaceutical companies to address the gaps in knowledge on the effects of drugs and medications on pregnancy and breastfeeding. The new Pregnancy and Lactation Labeling Rule makes the potential risks and benefits of drugs on pregnant and lactating women clearer and more comprehensive, replacing the previous letter categories of A, B, C, D, and X [81]. This rule encourages more conversation and decision-making when it comes to pregnant and lactating women choosing to take certain medications and will hopefully increase clinical lactation study participation by bringing more light to this issue. As we can see in Table 3, there are a multitude of antidepressants without any information on their excretion into breast milk. More research must be conducted to answer this gap in knowledge.

While the M/P ratio is an important parameter for drug transferability, the evaluation of the overall systemic exposure to the drug through breast milk depends on a multitude of maternal and infant factors, not on M/P ratio alone. Age and the maturity of the eliminating organs impact the infant’s clearance, which plays a role in determining the potential of the drug to be absorbed into the infant’s systemic circulation [14]. The frequency of breastfeeding or partial formula feeding may also impact drug absorption, as more frequent exposure through breastfeeding may lead to prolonged drug half-lives in the infant [14]. In addition to the infant’s development, maternal factors such as the phase of milk production and fat composition can influence the potential for drug transfer from breast milk to infant [14]. It is important to consider these additional factors when evaluating the safety of drug therapy in lactation.

## 15. Conclusions

The lack of clinical data and challenges associated with conducting clinical lactation trials warrant the use of alternative methods to evaluate the transfer of drug into human breast milk. Several methods, encompassing in vitro, in vivo, and in silico approaches, enable us to estimate the transfer of antidepressants into breast milk. In vitro cell cultures offer a means to predict M/P ratios without human subject involvement. Meanwhile, animal lactation models serve to evaluate drug transfer from a preclinical perspective. Additionally, in silico techniques like PBPK and QSAR modeling are increasingly viable for quantitatively determining drug transferability. The utilization of retrospective studies can also aid in assessing exposure outcomes. However, much remains unknown regarding the safety of numerous antidepressants. An integrated approach that combines various techniques, including in vivo, in vitro, ex vivo, and in silico methods, is essential to expedite the availability of pharmacology data for pregnant and lactating populations. 

## Figures and Tables

**Table 1:** Animal studies to determine antidepressant drug secretion into breast milk.

Drug	Drug Class	Animal Species	M/P Ratio	Reference
Agomelatine	Melatonergic and serotonergic agonist ^#^	Rat	0.348–1.128	EMA, 2008 [39]
Moclobemide	MAOI	Mouse	1.41	Ito et al., 2013 [40]
Trazadone	SARI	Mouse	0.20	Ito et al., 2013 [40]
Escitalopram	SSRI	Rat	<0.15 *	Bourke et al., 2011 [41]
Paroxetine	SSRI	Rat	<0.028 *	Bourke et al., 2011 [41]
Fluoxetine	SSRI	Rat	<0.028 *	Bourke et al., 2011 [41]
Venlafaxine	SNRI	Rat	<0.057 *	Bourke et al., 2011 [41]

* M/P ratio was not available. The listed value is Serum Conc Ratio (Pup/Dam)**,** which was calculated by using the drug concentration in the pup’s serum divided by that in the dam’s serum on day 7 of drug exposure. Median values for pup and dam serum on day 7 were averaged to obtain this value, and only data from litters 1 and 2 were utilized. ^#^ MAOI: Monoamine oxidase inhibitor; SARI: Serotonin agonist and reuptake inhibitor; SSRI: Selective serotonin reuptake inhibitor.

**Table 3:** Antidepressants without knowledge of secretion into breast milk.

Drug	Drug Class ^#^	First-Line/Second-Line Treatment	Lactation Adverse Events (If Reported)	Year Drug Approved
Agomelatine	Melatonergic and serotonergic agonist	Not available in the U.S.	Possible drowsiness and developmental concerns in one infant out of sixteen infants	Not approved in the U.S.
Isocarboxazid	MAOI	Second line	Unknown	1959
Levomilnacipran	SNRI	First line	Unknown	2013
Lofepramine	Tricyclic	Not available in the U.S.	Unknown	Not approved in the U.S.
Phenelzine	MAOI	Second line	Unknown	1961
Tranylcypromine	MAOI	Second line	Abdominal distention and feeding intolerance in one infant	1961
Trimipramine	Tricyclic	Available in the U.S. but not clinically used	Unknown	1982
Vilazodone	SPARI	First line	Unknown	2011
Vortioxetine	SPARI	First line	No adverse effects reported	2013

^#^ SPARI: serotonin partial agonist-reuptake inhibitor.

## Data Availability

The data that support the findings of this study are available from the corresponding author upon reasonable request.
